# Frontostriatal pathways gate processing of behaviorally relevant reward dimensions

**DOI:** 10.1371/journal.pbio.2005722

**Published:** 2018-10-19

**Authors:** Susanna C. Weber, Thorsten Kahnt, Boris B. Quednow, Philippe N. Tobler

**Affiliations:** 1 Zurich Center for Neuroeconomics, Department of Economics, University of Zurich, Zurich, Switzerland; 2 Department of Neurology, Northwestern University Feinberg School of Medicine, Chicago, Illinois, United States of America; 3 Experimental and Clinical Pharmacopsychology, Department of Psychiatry, Psychotherapy and Psychosomatics, Psychiatric Hospital, University of Zurich, Zurich, Switzerland; 4 Neuroscience Center Zurich, University of Zurich and Swiss Federal Institute of Technology Zurich, Zurich, Switzerland; University of Oxford, United Kingdom of Great Britain and Northern Ireland

## Abstract

The value of rewards arises from multiple hedonic and motivational dimensions. Reward-encoding brain regions such as the ventral striatum (VS) are known to process these dimensions. However, the mechanism whereby distinct reward dimensions are selected for neural processing and guiding behavior remains unclear. Here, we used functional imaging to investigate how human individuals make either hedonic (liking) or motivational (wanting) evaluations of everyday items. We found that the two types of evaluations were differently modulated depending on whether participants won or lost these items. Neural activity in the VS encoded both hedonic and motivational dimensions of reward, whereas ventromedial prefrontal activity encoded primarily motivational evaluations and central orbitofrontal activity encoded predominantly hedonic evaluations. These distinct prefrontal representations arose regardless of which judgment was currently relevant for behavior. Critically, the VS preferentially processed the reward dimension currently being evaluated and showed judgment-specific functional connectivity with the dimension-specific prefrontal areas. Thus, our data are in line with a gating mechanism by which prefrontal cortex (PFC)–VS pathways flexibly encode reward dimensions depending on their behavioral relevance. These findings provide a prototype for a generalized information selection mechanism through content-tailored frontostriatal communication.

## Author summary

People and animals typically both want and like rewards. Here, we show that these two dimensions of value can be dissociated at both the behavioral and the neural level. In keeping with rodent findings, our human neuroimaging data indicate that the ventral striatum—a part of the reward system in the basal ganglia—encodes both dimensions. However, it does so depending on the judgment being made: during wanting judgments, activity in the ventral striatum increases with the degree of wanting significantly more than with the degree of liking, and vice versa during liking judgments. Accordingly, activity in the ventral striatum expresses the value dimension currently needed for behavior. In contrast, distinct regions of the prefrontal cortex encode either the degree of wanting or the degree of liking, irrespective of judgment type. Functional coupling analysis suggests that the ventral striatum preferentially communicates with wanting- or liking-related regions in the prefrontal cortex according to the type of expressed judgment. These findings suggest that flexible frontostriatal coupling can serve a gating mechanism to achieve behaviorally relevant selection of value dimensions.

## Introduction

Reward is central for goal-directed behavior. However, reward is not a unitary concept but characterized by multiple dimensions. Activity in reward-processing regions such as the ventral striatum (VS) correlates with various reward dimensions, including gains and losses [1], pleasantness [2], hedonic value [3], motivational value [4,5], expected value [6,7], received value [8], decision value [9], and salience [10]. Some of these different reward dimensions can be separated at the behavioral level [11,12]. This raises an important yet unresolved question: does the VS process these dimensions simultaneously and in parallel, irrespective of which dimension is currently relevant for behavior? Alternatively, if the VS processes only one dimension at a time, how does the VS selectively and flexibly gate access to the behaviorally relevant signals?

Here, we focus on two common reward dimensions [13–15] that overlap anatomically in the VS [12,13,16]: the motivational drive to obtain rewards (wanting) and the hedonic pleasure associated with rewards (liking; please note that we use the terms “wanting” and “liking” in their everyday meaning, i.e., as measured by self-report [11,12]). We used a behavioral task in which participants indicated how much they wanted or liked various nonconsumable reward items, and we aimed to dissociate the motivational and hedonic reward dimensions by having participants win or lose these items in a game. Given the VS’s central position at the center of corticostriatal loops [17], the VS could participate in largely separate and parallel wanting and liking loops, passing on information received from distinct regions in the medial prefrontal cortex (mPFC) and orbitofrontal cortex (OFC). This possibility mirrors traditional views of cortical and basal ganglia architecture [18,19] and predicts that VS activity should scale with wanting or liking ratings irrespective of whether the current judgment is a wanting or a liking judgment. In contrast, based on the anatomical convergence of prefrontal projections in the VS [20,21], the VS could dynamically interact with cortical wanting and liking regions depending on which dimension is currently required for guiding behavior. In this view, VS activity should reflect primarily wanting ratings during wanting judgments and primarily liking ratings during liking judgments.

In line with the second mechanism, we find evidence compatible with the idea of striatal gating of hedonic and motivational reward dimensions. In contrast to the judgment-specific coding observed in the VS, distinct regions in the mPFC and OFC encoded wanting or liking regardless of judgment type. Finally, frontostriatal connectivity varied as a function of judgment type, supporting the idea that access to the currently relevant reward dimension is gated in the striatum.

## Results

### Wanting and liking judgments are differently affected by gains and losses

**Fig 1:**
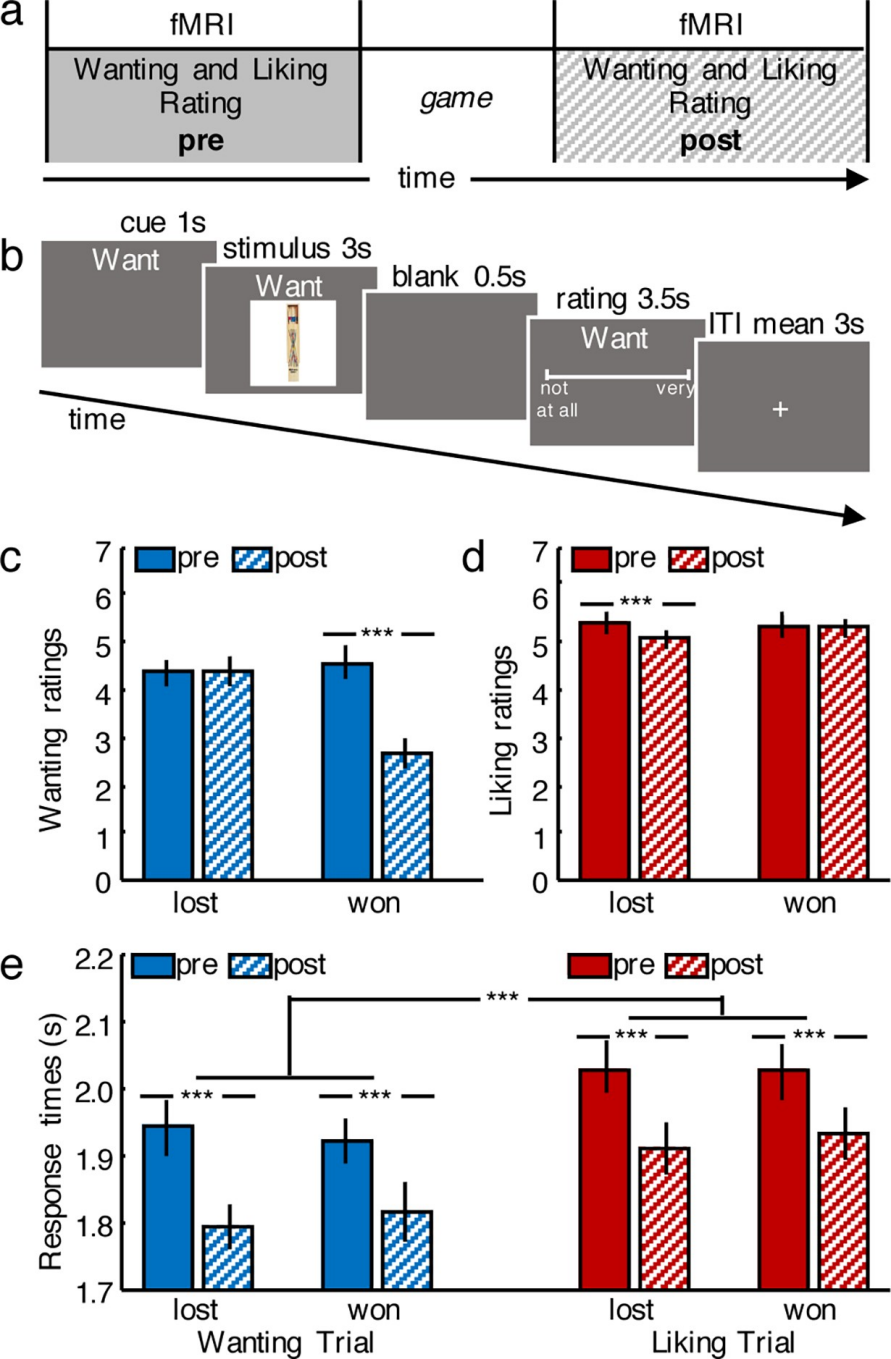
Task and behavior. **A.** Timeline of the experimental design. Wanting and liking ratings were collected in the scanner. After the initial session (pre), participants were removed from the scanner and completed a game on a computer in an adjacent room. Participants were then asked to rate the items in a second session (post) in the scanner. **B.** Timing of the scanned task. After a trial-type identifying cue, participants viewed an item (here, pick-up sticks game; picture taken by authors) and then judged it. Wanting and liking judgments, as well as the location of the anchor points of the rating scale, were randomized across trials. **C.** Change in wanting ratings as a function of game outcomes. Wanting decreased from pre- to postgame specifically for won items but remained similar for lost items (wanting won pre versus won post: t(27) = 4.81, *p* < 0.001; wanting lost pre versus lost post: t(27) = −0.16, *p* = 0.873). **D.** Change in liking ratings as a function of game outcomes. Liking decreased from pre- to postgame specifically for lost items but remained similar for won items (liking won pre versus won post: t(27) = 0.52, *p* = 0.609; liking lost pre versus lost post: t(27) = 4.50, *p* < 0.001). **E.** Response times for the ratings. Participants became significantly faster from pre- to postgame and took significantly longer to make liking judgments compared to wanting judgments. ***p < 0.001; error bars depict SEM. Data in S1 Data. fMRI, functional magnetic resonance imaging; SEM, standard error of the mean.

Participants rated everyday items in the scanner according to how much they wanted and how much they liked them (Fig 1A and 1B). The ratings in the scanner were collected twice—once before and once after participants played a game in which they won half of the items. Won items were handed over to participants at the end of the game. The game allowed us to separate wanting and liking behaviorally while also making the task more engaging.

Participants differentiated between wanting and liking judgments in terms of both response times and ratings (Fig 1C–1E). Analyzing response times using an ANOVA with repeated-measures factors Session (pre- or postgame), Judgment Type (wanting or liking rating), and Stimulus Type (won or lost item) revealed a main effect of Session (F(1,27) = 29.94, *p* < 0.0001), as well as a main effect of Judgment Type (F(1,27) = 41.10, *p* < 0.0001). Participants took significantly more time to make liking judgments than wanting judgments (t(27) = 6.39, *p* < 0.001; Fig 1E), and response times correlated (positively) with ratings only for wanting (r = 0.33, *p* = 0.04) but not for liking (r = −0.09, *p* = 0.56) judgments. Together, these findings suggest that participants treated the two judgment types differently.

Furthermore, even though they remained significantly correlated overall (before game: r = 0.79; after game: r = 0.78, both *p* < 0.001), wanting and liking ratings changed differentially from before to after the game depending on whether the item was lost or won. An ANOVA served to analyze the change in ratings, with repeated-measures factors Judgment Type (wanting or liking rating) and Stimulus Type (won or lost item). We found both main effects of Judgment Type (F(1,27) = 10.49, *p* < 0.005) and Stimulus Type (F(1,27) = 21.40, *p* < 0.0001), as well as an interaction between Stimulus and Judgment Type (F(1,27) = 34.50, *p* < 0.0001). Wanting ratings decreased specifically for won items (change in wanting won versus lost items: t(27) = −5.28, *p* < 0.001; wanting won pre versus won post: t(27) = 4.81, *p* < 0.001; wanting lost pre versus lost post: t(27) = −0.16, *p* = 0.873; Fig 1C). In contrast, liking ratings decreased specifically for lost items (change in liking won versus lost items: t(27) = 2.79, *p* < 0.05; liking won pre versus won post: t(27) = 0.52, *p* = 0.609; liking lost pre versus lost post: t(27) = 4.50, *p* < 0.001; Fig 1D). Taken together, these differences in response times and ratings provide evidence that the participants differentially processed the hedonic and motivational dimension of items.

### Neural activity in the OFC and mPFC correlates with either wanting or liking

**Fig 2:**
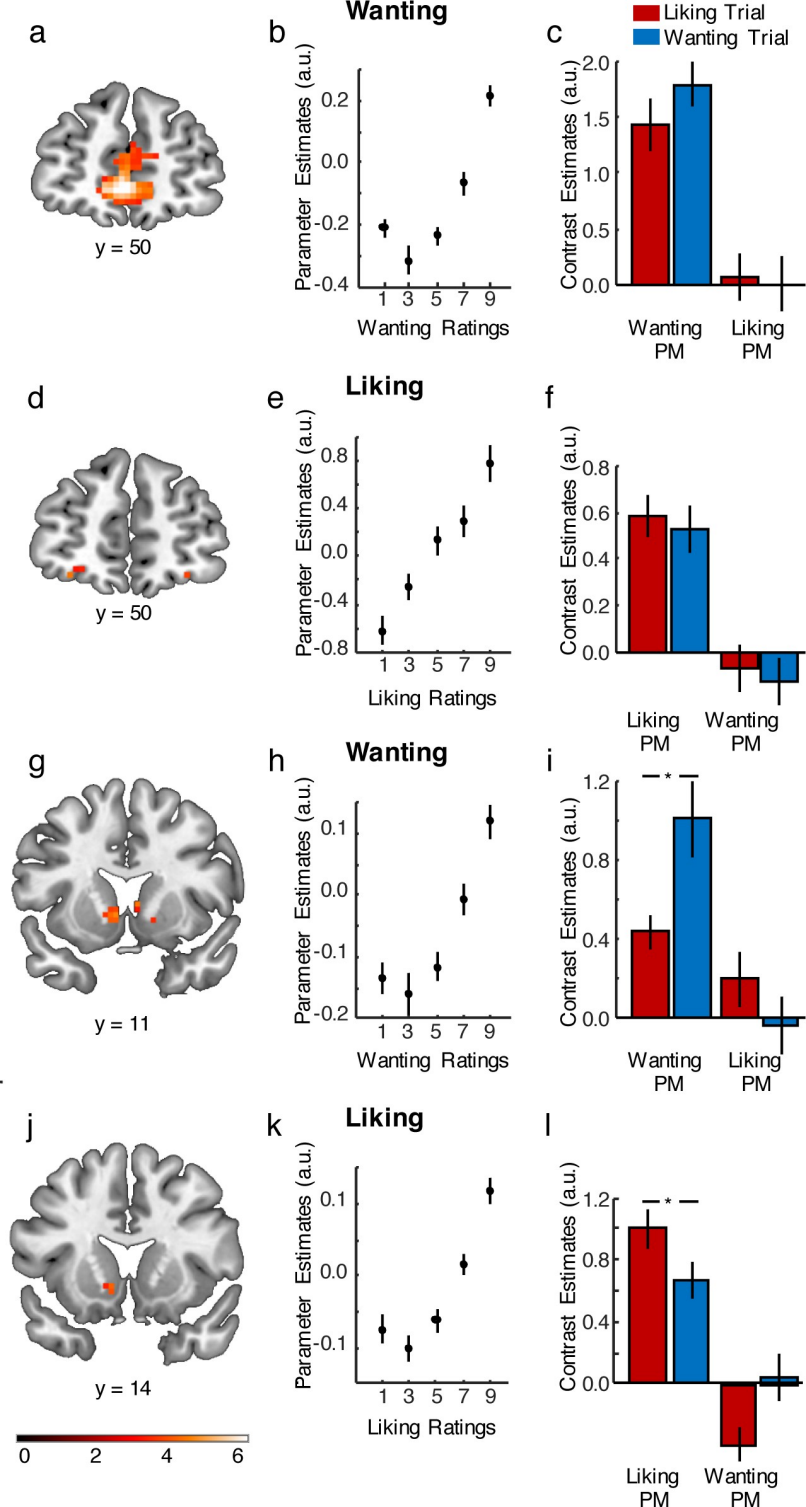
Neural encoding of motivational and hedonic reward dimensions. **A–B.** Wanting ratings correlated with activity in the mPFC. **C**. Contrast estimates for the wanting and liking PMs show that correlation of mPFC activity with wanting ratings was stronger than with liking ratings and occurred irrespective of which judgment was expressed in behavior. **D–E.** Liking ratings correlated with activity in the central OFC. **F**. The correlation of central OFC activity with liking ratings was stronger than with wanting ratings and occurred irrespective of judgment type. **G–H**. Wanting ratings correlated with activity in the VS. **I**. Contrast estimates show stronger relation to wanting than liking PMs particularly during wanting trials. **J–K.** Liking ratings correlated with activity in the VS. **L.** Contrast estimates show stronger relation to liking than wanting PMs, particularly during liking trials. *p < 0.05; error bars depict SEM. Data in https://neurovault.org/collections/4266/ and S1 Data. mPFC, medial prefrontal cortex; OFC, orbitofrontal cortex; PM, parametric modulator; SEM, standard error of the mean; VS, ventral striatum.

We next assessed which neural systems encoded wanting and liking. Using a parametric general linear model (GLM), we identified regions where activity was parametrically associated either with wanting or with liking ratings (Table 1 and Fig 2). In this GLM, we pooled data from both liking and wanting trials, resulting in one onset regressor, which was modulated by three parametric modulators (PMs): the individual average wanting rating of the presented item, the individual average liking rating of the presented item, and the trial-specific response time (serial orthogonalization of parametric regressors was turned off for these analyses [22]). In a whole-brain (voxel-level) corrected analysis, we found that wanting was related to prefrontal activations, including medial parts of the OFC (z = 5.03, family-wise error (FWE)-corrected, *p* < 0.05, peak [0, 50, −5]; Fig 2A), and the mPFC (z = 5.21, FWE-corrected, *p* < 0.05, peak [−3, 44, −2]). In contrast, liking-related responses were more focal and limited to the central OFC (z = 4.86, FWE-corrected, *p* < 0.05, peak [−24, 47, −14]; Fig 2D) and posterior cingulate (z = 4.92, FWE-corrected, *p* < 0.05, peak [0, −34, 25]). These results suggest that neural activity in anatomically segregated regions of the prefrontal cortex (PFC) track either wanting or liking.

**Table 1:** Brain regions associated with liking or wanting irrespective of judgment type.

		MNI Coordinate				
	Region	*x*	*y*	*z*	*T*	*k* voxels
**Liking**	Central OFC	−24	47	−14	6.23*	11
	Posterior cingulate	0	−34	25	6.34*	250
	VS	−9	14	−5	4.41	6
	Pallidum	−15	5	−2	4.69	2
**Wanting**	Medial OFC	0	50	−5	6.56*	180
	mPFC	−3	44	−2	6.93*	356
	left VS	−6	11	−2	4.83	22
	right VS	6	11	4	4.63	7
		12	14	−11	4.27	3

Results surviving voxel-wise FWE-correction for multiple comparisons.

* indicates *p* < 0.05 corrected for multiple comparisons across the whole brain, all other regions significant after SVC; cluster size *k* based on *p* < 0.001 uncorrected threshold. Abbreviations: FWE, family-wise error; MNI, Montreal Neurological Institute; mPFC, medial prefrontal cortex; OFC, orbitofrontal cortex; SVC, small volume correction; VS, ventral striatum.

To further characterize the degree to which these responses are specific to wanting or liking judgments, we employed two post hoc region-of-interest (ROI) analyses. First, we extracted individual liking- and wanting-related responses in the ROIs associated with wanting and liking ratings (6 mm spheres around the peak voxels; Table 1) and assessed the difference between these responses. To minimize bias, the ROIs were defined using data from all subjects except the one for whom the neural responses were being extracted (leave-one-subject-out cross-validation procedure). This allowed us to determine whether different regions encoded wanting and liking differently or similarly. While wanting- and liking-related responses in the posterior cingulate ROI did not differ significantly (t(27) = 1.66, *p* = 0.108), those extracted from the OFC and mPFC ROIs did. Responses in the central OFC showed significantly stronger associations with liking than wanting (t(27) = 2.35, *p* = 0.026). In contrast, the medial OFC cluster as well as the mPFC cluster showed stronger responses for wanting than liking (medial OFC: t(27) = −2.07, *p* = 0.048; mPFC: t(27) = −1.99, *p* = 0.056). Second, we performed an ROI analysis with entirely independent ROIs from a meta-analysis of reward activity in the medial and lateral OFC [23]. This analysis yielded similar findings as the previous one: main effects of PM Type (F(1,27) = 4.59, *p* = 0.034) and ROI (F(1,27) = 12.43, *p* < 0.001) and a significant interaction of PM Type with ROI (F(1,27) = 8.90, *p* = 0.004). Pairwise comparisons showed significant coding of wanting (t(27) = 5.35, *p* < 0.001) but not liking (t(27) = 1.62, *p* = 0.116) and stronger coding of wanting than liking (t(27) = 2.53, *p* = 0.018) in the medial OFC. Conversely, the central OFC showed significant coding of liking (t(27) = 2.92, *p* = 0.007) but not wanting (t(27) = 1.20, *p* = 0.239), although the difference between liking and wanting (t(27) = 0.95, *p* = 0.349) was not significant. Together, these data suggest that wanting and liking tend to be processed in anatomically distinct regions in the PFC but overlap in the posterior cingulate.

### Neural activity in overlapping regions of the VS correlates with both wanting and liking

Previous animal work has implicated the VS (nucleus accumbens) and the pallidum in encoding both motivational and hedonic reward dimensions [24]. Based on these findings, we examined the role of these two areas in more detail. We analyzed data in two *a priori* anatomically defined ROIs encompassing these two regions (Table 1, Fig 2). In the pallidum, activity was parametrically associated only with liking ratings (z = 3.97, FWE-small volume correction (SVC), *p* < 0.01, peak [−15, 5, −2]). In the VS, we found parametric wanting-related activations (z = 4.06, FWE-SVC, *p* < 0.01, peak [−6, 11, −2]; Fig 2G), as well as more confined parametric liking-related activations (z = 3.79, FWE-SVC, *p* < 0.05, peak [− 9, 14, −5]; Fig 2J). Thus, in line with previous animal studies, the VS encoded both wanting and liking, whereas the pallidum processed primarily hedonic evaluations.

**Fig 3:**
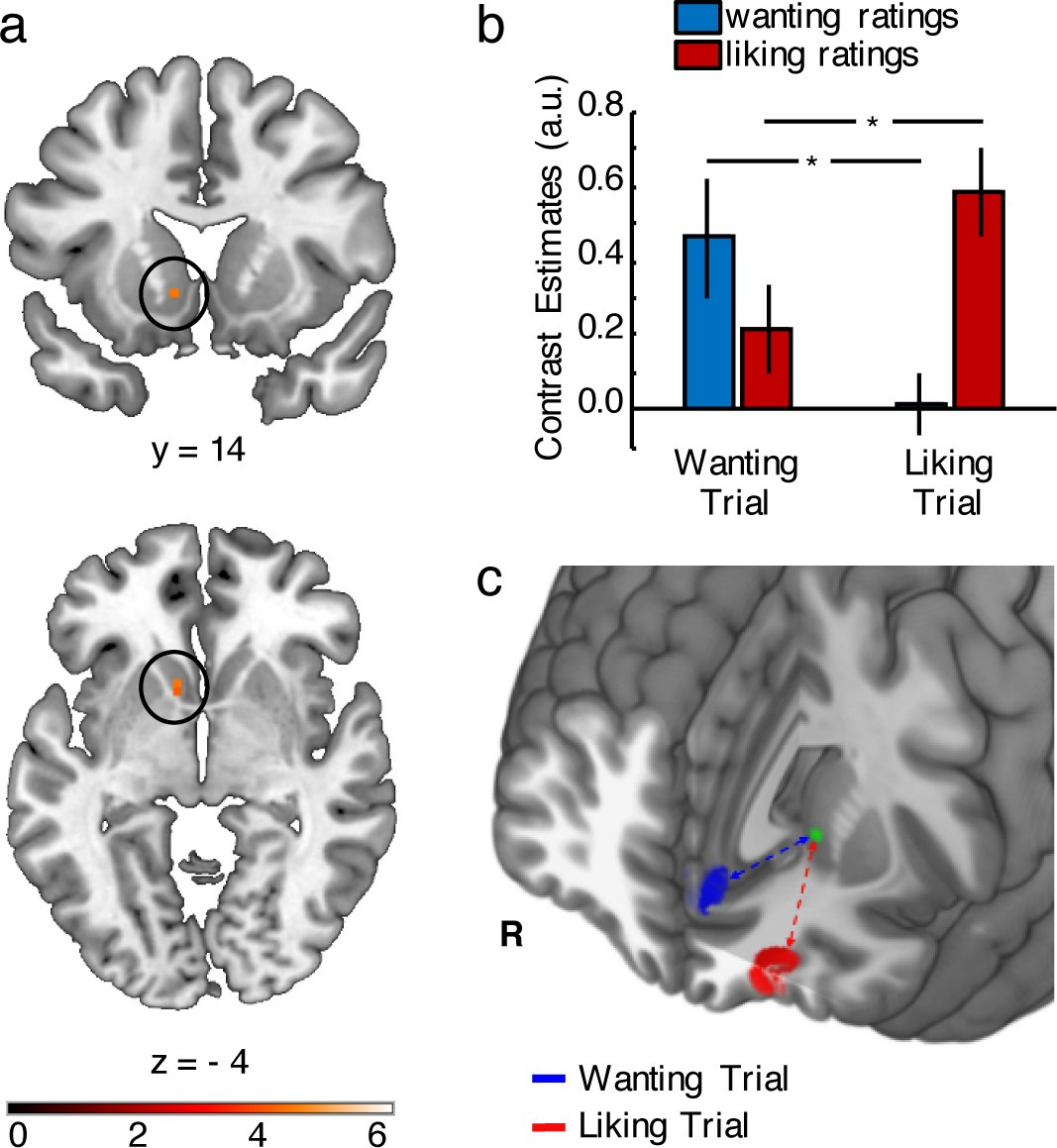
Potential gating of behaviorally relevant reward dimensions by frontostriatal connectivity. **A–B**. Behaviorally relevant encoding of wanting or liking levels in the VS. **A.** Conjunction of the PMs for wanting and liking. **B.** Activity in the VS encoded wanting ratings during wanting trials and liking ratings during liking trials. **C.** Functional connectivity between VS and prefrontal activations related to current wanting and liking levels depended on whether participants were making wanting or liking judgments. *p < 0.05; error bars depict SEM. Data in S1 Data. R, right; SEM, standard error of the mean; VS, ventral striatum.

To more systematically assess the relation of these striatal and pallidal responses to wanting and liking, we extracted and compared both wanting- and liking-related responses from 6 mm sphere ROIs in the VS and pallidum, again using a leave-one-subject-out cross-validation procedure (Table 1). In contrast to the PFC clusters, comparable wanting- and liking-related responses were found in the VS ROI associated with liking (t(27) = −0.50, *p* = 0.622) as well as the VS ROI associated with wanting (t(27) = −1.02, *p* = 0.315). While the pallidum ROI associated with liking showed no difference to wanting (t(27) = −1.12, *p* = 0.906), it is worth keeping in mind that we found no significant relation to wanting in the pallidum to start with. In line with an overlap of both reward dimensions primarily in the VS, a formal conjunction analysis [25] revealed common wanting and liking areas in the VS (z = 3.97, FWE-SVC, *p* < 0.05, peak [− 9, 11, −5]; Fig 3A) but not in the pallidum and the posterior cingulate. Thus, while prefrontal responses appear to be specific to either wanting or liking and exhibit a regional dissociation between the two, responses in the VS (and to a lesser degree in the pallidum and posterior cingulate) seem to encode both reward dimensions.

### Main effects of the game

To investigate the effects of game outcome, we assessed mean neural activity elicited by item onset (irrespective of trial-specific rating) in exploratory analyses of the ROIs identified by the parametric analyses reported above. Central OFC activity decreased more for lost than won items (t(27) = 2.60, *p* = 0.015) and mPFC activity decreased more for won than lost items in wanting trials (t(27) = 2.83, *p* = 0.009). Finally, the VS showed decreases in activity for both won and lost items (t(27) > 3.77, *p* < 0.001). These findings are consistent with coding of mean behavioral liking decreases by central OFC, mean behavioral wanting decreases by mPFC, and coding of both of these effects by the VS.

### Striatum but not PFC encodes reward dimensions depending on behavioral relevance

The results reported above suggest that wanting and liking are encoded in overlapping regions in the striatum but in separate regions in the PFC. We next assessed whether encoding of these two dimensions in the VS depends on which dimension is currently relevant for behavior. We therefore tested whether the responses identified by the parametric GLM were independent of the type of judgment participants made in a given trial or whether the VS switched between coding wanting and liking as a function of judgment type. For this analysis, we used a second parametric GLM that distinguished between trials with different judgement types (two regressors corresponding to trials in which liking and wanting judgments were made, respectively). Each of these regressors was again parametrically modulated by the individual average wanting rating of the presented item, the individual average liking rating of the presented item, and the trial-specific response time (serial orthogonalization of parametric regressors was again turned off for these analyses [22]). These analyses were performed in ROIs of 6 mm spheres around the peak voxels from the first parametric GLM (Table 1). We extracted and compared wanting-related responses during wanting and liking trials as well as liking-related responses during wanting and liking trials. This allowed us to assess whether responses were specific to the currently performed judgment (e.g., for wanting, specificity would be reflected in significantly stronger encoding of wanting ratings during wanting judgments compared to liking judgments).

For both liking- and wanting-related responses, areas in the PFC and posterior cingulate encoded reward dimensions irrespective of judgment type. Specifically, we found that liking-related responses within the central OFC ROI were significant during both liking and wanting judgments (liking trials: t(27) = 2.83, *p* = 0.009; wanting trials: t(27) = 2.15, *p* = 0.041) and did not differ significantly between judgment types (liking versus wanting trials: t(27) = 0.45, *p* = 0.655). Likewise, liking-related responses in the posterior cingulate were significant during both judgment types (liking trials: t(27) = 4.41, *p* = 0.0001; wanting trials: t(27) = 4.14, *p* = 0.0003) and did not differ significantly (liking versus wanting trials: t(27) = 0.23, *p* = 0.823). Moreover, wanting-related responses in the mPFC and medial OFC were significant during both wanting and liking trials and did not differ significantly between judgment types (mPFC: wanting trials t(27) = 4.83, *p* = 0.00005; liking trials t(27) = 4.57, *p* = 0.0001; wanting versus liking trials t(27) = 0.12, *p* = 0.903; medial OFC: wanting trials t(27) = 5.33, *p* = 0.00001; liking trials t(27) = 4.15, *p* = 0.0003; wanting versus liking trials t(27) = 0.51, *p* = 0.613). Thus, beyond exhibiting regional specificity for motivational versus hedonic reward dimensions, these anatomically segregated cortical regions also appear to consistently track wanting or liking regardless of which judgment is currently being made.

In contrast, responses in the VS strongly depended on the current judgment type. Parametric liking-related responses in the VS were only significant during liking judgments (liking trials: t(27) = 4.85, *p* = 0.00005; wanting trials: t(27) = 1.49, *p* = 0.15) and significantly stronger during liking than wanting judgments (liking versus wanting trials: t(27) = 2.32, *p* = 0.028). Conversely, parametric wanting-related responses in the VS were only significant during wanting judgments (wanting trials: t(27) = 3.61, *p* = 0.001; liking trials: t(27) = 1.27, *p* = 0.216) and significantly stronger for wanting than liking judgments (wanting versus liking trials: t(27) = 2.80, *p* = 0.009). Focusing on the activation pattern of the common overlapping voxels in the VS (Fig 3A) mirrored this finding. We compared wanting-related and liking-related signals in the VS cluster defined by the conjunction analysis using an ANOVA with repeated-measures factors Judgment Type (wanting or liking trial) and PM Type (wanting or liking). In line with selective processing of the currently relevant reward dimension, we observed a significant interaction (F(1,27) = 7.17, *p* = 0.012; Fig 3B). Specifically, the VS showed stronger parametric wanting-related responses during wanting judgments than liking judgments (t(27) = 2.53, *p* = 0.018) and stronger parametric liking-related responses during liking than wanting judgments (t(27) = 2.28, *p* = 0.031). Taken together, while the frontal ROIs (OFC and mPFC) exhibit regional specificity for wanting and liking regardless of judgment type, the striatum flexibly encodes wanting or liking depending on whether wanting or liking judgments are required.

These findings imply that VS activity is closer to behavioral responses than central OFC and mPFC activity. To directly test this prediction, we extracted subject-wise time series from the VS, mPFC, and central OFC, z-scored them, and used them to predict trial-wise ratings irrespective of judgment type. The participant-specific regression model also included motion parameters. We then used paired *t* tests to compare the mean regression coefficients between brain regions. We find that VS activity is a significantly better predictor of trial-by-trial ratings than activity in mPFC (t(27) = 5.97, *p* = 0.000003) or central OFC (t(27) = 2.47, *p* = 0.02). These data corroborate the notion that VS activity is closer to behavior than medial prefrontal and central orbitofrontal activity.

### Frontostriatal pathways gate behaviorally relevant reward dimensions

Lastly, we explored the mechanism by which activity in the VS switched between encoding of different reward dimensions. One possible mechanism could be to flexibly enhance the cross-talk between the VS and the cortical region that processes the currently relevant dimension proportional to the current level of this reward dimension. To examine this possibility, we performed a psychophysiological interaction (PPI) analysis and tested whether functional coupling (fMRI signal coherence) between the VS and wanting and liking regions in the PFC depended on the type and level of the current judgment. We used the overlapping voxels in the VS as a seed region to extract the physiological signal. Psychological factors were liking and wanting judgment trials, each parametrically modulated by the average wanting and liking ratings of the current item. The PMs were multiplied by the physiological variable to generate a total of four psychophysiological regressors (liking-trial liking rating, liking-trial wanting rating, wanting-trial liking rating, wanting-trial wanting rating). As target regions, we focused on the same ROIs in the central OFC and mPFC defined above that processed wanting and liking ratings irrespective of the current judgment. During liking judgments, we found that VS connectivity with the central OFC was more strongly related to levels of liking than levels of wanting (z = 3.26, FWE-SVC, *p* < 0.05, peak [−21, 44, −11]; Fig 3C). Conversely, during wanting judgments, we found that VS connectivity with the mPFC was more strongly related to levels of wanting than levels of liking (z = 3.10, FWE-SVC, *p* < 0.05, peak [−6, 44, 4]; Fig 3C). Together, these results suggest that flexible processing of reward dimensions in the VS may be realized by selectively gating input from prefrontal regions that encode the reward dimension that is currently relevant for behavior. However, it should be kept in mind that a gating mechanism is only one possible interpretation of our functional coupling data. In any case, the degree of this connectivity modulation is directly related to the level of the currently relevant reward dimension.

## Discussion

A key contribution of our study is to clarify the role of the striatum in processing different dimensions of reward. We found that the striatum, in contrast to prefrontal regions, flexibly encodes reward dimensions depending on which dimension is currently relevant for behavior. This provides important insight into how reward information may be transformed in cortico-striatal circuits. The functional and anatomical nature of these circuits has been the focus of substantial amount of research. While earlier animal studies had suggested mainly segregated, independent, and parallel processing of information [26,27], recent models of how information is processed in corticostriatal loops have proposed a more integrative role for the striatum in which information from the cortex converges in the striatum and only behaviorally relevant information is passed on [28–32]. Our results support the latter scheme because we demonstrate that one common VS region encodes the currently relevant reward dimension.

In contrast to overlapping coding of wanting and liking in the VS, we find anatomical specificity in encoding of reward dimensions irrespective of behavioral relevance in the PFC. Thus, the PFC appears to process reward dimensions in a segregated and parallel manner. Specifically, we demonstrate that the motivational aspect of reward is processed by medial parts of the OFC, while the hedonic aspect is processed by the central/lateral OFC. A similar medial–lateral distinction has been observed in prior animal recording and human imaging studies, with medial frontal regions exhibiting a role in goal-directed decision processes [33,34] and lateral frontal regions being more strongly involved in encoding emotion and affective values of specific outcomes [34–39]. Our results extend this literature by demonstrating not only that areas of the PFC are anatomically segregated in function but also that they process reward information in a parallel and consistent manner, irrespective of the current behavioral requirements.

Our findings are in line with the notion that information about distinct reward dimensions is segregated in the cortex, then converges onto the striatum and is expressed there according to which type of value judgment is required. They inform current models of basal ganglia function and suggest how the basal ganglia selects appropriate actions while facing considerable convergence of cortical information [28,32]. Our data also suggest that flexible changes in VS encoding of reward dimensions are mediated by changes in regionally corresponding allocation of frontostriatal connectivity, with the strength of VS connectivity with specific regions in PFC being directly related to the level of the currently processed reward dimension. This is neurobiologically plausible, as the striatal spiny neurons receive input from numerous cortical neurons and can use pattern recognition to detect what is currently behaviorally relevant to the individual [21,29,30,40]. In fact, behaviorally specific striatal single-unit activity has been demonstrated for motor programs [41]. Mechanistically, the striatal spiny neurons could signal behaviorally relevant cortical value input, which could lead to a pause in firing in the pallidum and in turn produce specific activity for appropriate initiation of an action. Additionally, striatal dopamine may support the gating and controlling of cognitive representations from the PFC [42]. Together, our data suggest a mechanism for how the striatum selects behaviorally relevant information by gating cortical inputs.

Our findings of common wanting and liking signals in the VS are in line with numerous animal studies investigating hedonic and motivational reward dimensions [3,24,43–45]. Similarly, human neuroimaging studies using dietary restraint and satiation have found both wanting and liking signals in the VS [46,47]. In light of these studies, our current finding of relevance-dependent encoding of wanting in the VS suggests that it would be worthwhile to investigate whether and how the behavioral relevance of wanting judgments for food rewards is modulated by satiety and dietary restraint.

Cortical reward signals have been linked to both hedonic and motivational dimensions of reward. In rats, Mena and colleagues [48] found that local administration of a mu-opioid receptor agonist in the OFC and mPFC (corresponding roughly to the infralimbic and prelimbic cortex) led to increased food intake. In humans, the OFC is often identified as an important reward and pleasure center, with medial and central parts of the OFC responding to pleasant tastes and smells [38,49] and to monetary [50] and implicit and explicit social rewards [51,52], as well as to pleasant musical chords [53]. Particularly, medial and more dorsal regions of the OFC extending into the anterior cingulate and mPFC have also been associated with processing decision value [38,39,54,55], which is directly related to how much a choice alternative is wanted [56,57]. In line with this view, we and others find wanting signals in the medial OFC [47] as well as ventral parts of the mPFC [58–60]. More importantly, we go beyond previous findings by revealing that wanting-related parametric value levels activate the mPFC more than liking-related value levels and thereby specify the function of this core component of the valuation system.

Finally, dysfunctions in frontostriatal loops are implicated in several neuropsychiatric disorders such as obsessive–compulsive disorder, addiction, and schizophrenia. In particular, addiction could be viewed as a wanting-dominated state [12] in which behaviorally appropriate switching to liking no longer works. Our results raise the possibility that altered frontostriatal coupling contributes to such switching deficits.

It is worth noting several limitations of our study. First, we studied motivational and hedonic judgments, which are not fully equivalent with wanting and liking. In the animal literature [13–15], wanting is typically operationalized as approach behavior, whereas liking is captured with orofacial expressions associated with consuming a good. Thus, in the animal literature, the behavioral relevance of a wanting signal may be higher than that of a liking signal, although it should be kept in mind that orofacial expressions are also a form of behavior. In contrast, we operationally and more artificially define “behaviorally relevant” in our paradigm with expressing one judgment rather than another through rating. It therefore remains to be seen to what degree our results generalize to more ecological situations for which the studied brain regions have evolved. Previous research showed that liking ratings in the lab predict future consumption choice in other environments [61] and ratings for snack foods in the field [62], in line with at least some ecological generalizability. In any case, reducing the behavioral asymmetry between judgment types and using nonconsumable outcomes in our paradigm allowed us to elicit motivational and hedonic evaluations without introducing major task differences and thus to avoid visual, cognitive, motor, and other confounds. Second, it is unlikely that the entire affective experience related to a given good is captured by the liking rating. However, the use of an explicit rating allowed us to circumvent the issue (e.g., [13–15]) that objective behavioral measures of liking (e.g., tongue protrusions) cannot be taken as a proxy of an evaluative judgment in the absence of a (rating-like) subjective report. Third, VS and mPFC activity showed a trend for a quadratic relation between activity and ratings. This pattern could indicate that the observed activity partly reflects confidence, which is known to be higher for more extreme ratings or easier choices and to activate ventral parts of the mPFC [63,64]. Higher confidence is typically associated with faster responding [63,64]. Note, though, that we identified a dissociation between hedonic and motivational evaluations using linear rather than quadratic parametric modulation and that we accounted for response times by including them into our GLM. Moreover, the observation that brain activity but not response times showed quadratic trends indicates that confidence did not play a prominent role in the present paradigm. Finally, even though we made an effort to uncouple hedonic from motivational evaluations, we succeeded only partially. Indeed, in everyday life, the two judgment types are tightly coupled such that we typically like what we want and want what we like. This may also explain why some research reported mPFC activity in tasks that considered only hedonic evaluations or pleasantness ratings, which may capture aspects of both dimensions [65–67].

### Conclusions

We find anatomically segregated wanting- and liking-related signals in the PFC, as well as overlapping wanting- and liking-related responses in the VS. Our results are consistent with the idea that hedonic and motivational reward dimensions from the cortex converge in the striatum and are passed on from the striatum in a condensed and focused manner. We propose that this selection process is mechanistically implemented through frontostriatal gating of different reward signals. In the PFC, motivational and hedonic dimensions of reward are encoded in a parallel and anatomically separated manner, while the VS flexibly encodes only the reward dimension that is currently relevant for behavior. Thereby the striatum acts as a detector for behaviorally relevant reward dimensions and enables selective processing of reward information required for guiding ongoing actions appropriately. Thus, our findings show how the VS reduces the multiplexed nature of reward information and enables adaptive action selection. More generally, we demonstrate that besides selecting actions that provide the highest (decision) value within a given situation, the brain can also contextually select value representations. Finally, our data suggest situation-adapted modulation of connectivity as one possibility of achieving selection.

## Materials and methods

### Ethics statement

All participants provided informed written consent. The study complied with the Declaration of Helsinki and was approved by the ethics committee of the Canton of Zurich (protocol 2010-0327/3).

### Participants

We studied 28 right-handed participants aged 20–29 years (22.8 ± 0.5 years, mean ± SEM; 14 females). All participants were recruited from the Laboratory for Social and Neural Systems Research participant pool.

### Design and procedure

Forty nonconsumable everyday items were used as rewards in the study (S1 Table for a full list). Items were selected based on prior pilot experiments so that initial mean liking and wanting ratings were similar. Before scanning, we physically presented all items to participants in real life, which ensured that they recognized and were familiar with each item. Moreover, participants learned to separately consider hedonic and motivational dimensions of a good that they did not possess, such as an expensive sports car. The task was implemented with Matlab (The MathWorks, Natick, MA, United States) and the Cogent 2000 toolbox (http://www.vislab.ucl.ac.uk/cogent.php).

In the scanner, participants were asked to rate each item according to how much they wanted to have it, as well as how much they liked the item at that moment. In each trial (Fig 1B), participants first saw a cue indicating the type of rating trial (1 s), followed by an image of the item (3 s), and finally the rating screen (3.5 s). Ratings were provided on a continuous scale using a trackball. Trials were separated by a variable intertrial interval (mean 3 s). Each item was rated twice for wanting and twice for liking, resulting in 160 trials split into 4 runs before the game and the same again after the game.

Participants performed the rating task in two sessions, which were separated by a game in which participants could win the items outside of the scanner (Fig 1A). The game consisted of a perceptual task in which participants had to indicate whether the item was presented to the left or the right of the midpoint of the screen. Participants won items that they classified correctly. The difficulty of the game was calibrated so that participants won and lost 50% of the items. To make the items more salient and thereby enhance the memorability of winning and losing the items, participants were seated at a table with the items set up next to them while they performed the task on a computer. Additionally, immediately after the game, participants packed up the items they won in a bag, which they later took home.

### MRI data acquisition

Whole-brain scanning was performed with a Philips Achieva 3T whole-body MRI scanner equipped with an 8-channel head coil (Philips, Amsterdam, the Netherlands). For each of the 8 scanning runs, 227 T2*-weighted whole-brain EPI images were acquired in ascending order (33 transverse [axial] slices per volume, field of view 192 mm × 192 mm × 108 mm, slice thickness 2.6 mm, 0.7 mm gap, in-plane resolution 2 mm × 2 mm, matrix 96 × 96, repetition time [TR] 2,000 ms, echo time [TE] 25 ms, flip angle 80°). Additionally, a T1-weighted turbo field echo structural image was acquired in sagittal orientation for each participant with the same angulation as applied to the functional scans (181 slices, field of view 256 mm × 256 mm × 181 mm, slice thickness 1 mm, no gap, in-plane resolution 1 mm × 1 mm, matrix 256 × 256, TR 8.4 ms, TE 3.89 ms, flip angle 8°).

### MRI preprocessing

Preprocessing and statistical analysis of the MRI data were performed using SPM8 (http://www.fil.ion.ucl.ac.uk/spm; Wellcome Trust Centre for Neuroimaging, London, United Kingdom). All EPI images were temporally corrected to the middle slice, realigned to the mean image, normalized (resampling to 3 mm × 3 mm × 3 mm voxels) to the standard EPI template of the Montreal Neurological Institute (MNI), and smoothed using a Gaussian kernel with 4 mm full width at half maximum (FWHM). We chose a relatively small smoothing kernel because we were particularly interested in the VS, and a recent meta-analysis found that in order to avoid bias against subcortical activations, applying minimal smoothing is recommended [68].

### MRI data analysis

To detect activity related to wanting or liking, we used a parametric analysis. The first GLM pooled data from wanting and liking judgments into one judgment-type–unspecific regressor, time locked to the onset of each trial. This regressor was modulated by 3 PMs: within-session normalized item-specific average wanting ratings, within-session normalized item-specific average liking ratings, and response times. Importantly, to ensure that all regressors explain only independent components of variance, serial orthogonalization of parametric regressors (as implemented in SPM) was turned off [22]. Moreover, the GLM contained the 6 nuisance movement parameters. The duration of the onset regressor was 7 s, which corresponds to the time participants had to view and rate each image (Fig 1B). We report whole-brain results (*p* < 0.05, voxel-level FWE corrected) as well as activations in the a priori ROIs, VS, and pallidum (*p* < 0.05, voxel-level FWE corrected). The VS ROI was based on earlier studies and included the nucleus accumbens, ventral caudate nucleus, and putamen rostral to the anterior commissure [69]. The pallidum ROI was derived from the automatic anatomical labeling (AAL) atlas incorporated in the WFU-PickAtlas Tool in SPM [70,71].

To determine whether responses were specific or common to wanting and liking, we used an ROI analysis. We checked for specificity by extracting parameter estimates for each of the wanting and liking ROIs identified in the parametric contrast and using paired *t* tests that determined whether parameter estimates of one PM were significantly higher than those of the other PM. In order to minimize bias, we used two approaches. First, we performed a leave-one-subject-out cross-validation procedure, in which we extracted the neural data for each subject from ROIs consisting of 6 mm spheres around the peak of the activations identified by a group analysis in which this subject was left out. By iterating over all participants, this allowed us to extract relatively unbiased parameter estimates for all participants. Second, we performed the analysis in entirely independent 6 mm spheres centered on coordinates reported by a meta-analysis of reward activity in the medial (4, 54, −4) and lateral (−18, 40, −16) OFC [23]. To determine common areas of wanting and liking, we used an inclusive masking procedure, which identifies areas significantly associated with both wanting and liking PMs [25].

We used a second GLM to investigate judgment-specific and judgment-unspecific activations. In this model, we separated wanting and liking trials so that there were two onset regressors corresponding to judgment type (wanting trial or liking trial), each of which had three PMs associated with it (within-session normalized average wanting ratings of the presented item, within-session normalized average liking ratings of the presented item, and trial-specific response times), as well as the six nuisance movement parameters. Again, serial orthogonalization of parametric regressors was turned off. We then used an ROI analysis to investigate whether responses to wanting and liking identified by the first GLM depended on judgment type. ROIs were 6 mm spheres around the peak of the activations identified by the first GLM. We used Marsbar [72] (http://marsbar.sourceforge.net/) to extract parameter estimates for each of the PMs split by judgment type, which were then tested using repeated-measures ANOVAs and paired *t* tests.

### Connectivity analysis

We performed a PPI analysis [73] with the VS (showing common coding of wanting and liking) as the seed region and Judgment type (wanting versus liking) and Level (parametric regressors for wanting versus liking ratings) as psychological factors. We used the generalized form of the PPI model [74] to test whether the strength of the functional connectivity between the VS and the cortical regions showing specific coding of either wanting or liking depended on the type and level of the judgment performed on a given trial. The seed region was defined by the overlap of the wanting- and liking-related activations (Fig 3A). For each subject, we estimated a PPI model with the activity in the seed region included as the physiological regressor and Judgment type (wanting trial or liking trial), modulated by the within-session normalized item-specific average wanting ratings, as well as the within-session normalized item-specific average liking ratings included as the psychological regressors. The four PMs were multiplied with the physiological variable to create the psychophysiological regressors of interest (liking-trial liking rating, liking-trial wanting rating, wanting-trial liking rating, wanting-trial wanting rating). The two critical comparisons of the PPI regressors were: wanting rating versus liking rating during wanting trials and liking rating versus wanting rating during liking trials. Please note that because the PPI model included the psychological and parametric rating regressors, any rating-level–dependent increases in connectivity are independent of the linear effects of these rating levels on activity. Thus, any significant interaction would show increased functional coupling between seed and other regions with increasing wanting/liking ratings rather than simple rating-induced activity changes in region pairs. We focused our analysis on the prefrontal clusters in the mPFC and OFC that were identified by the first GLM.

## Supporting information

S1 Table List of items used in the study. (DOC)

S1 Data Excel spreadsheet containing, in separate sheets, the underlying numerical data for Fig 1C, 1D and 1E; Fig 2B, 2C, 2E, 2F, 2H, 2I, 2K and 2L; and Fig 3B. (XLSX)

## Abbreviations

AAL: automatic anatomical labeling
fMRI: functional magnetic resonance imaging
FWE: family-wise error
FWHM: full width at half maximum
GLM: general linear model
MNI: Montreal Neurological Institute
mPFC: medial prefrontal cortex
OFC: orbitofrontal cortex
PFC: prefrontal cortex
PM: parametric modulator
PPI: psychophysiological interaction
ROI: region of interest
SVC: small volume correction
TE: echo time
TR: repetition time
VS: ventral striatum
