# Supplementary material for: Frontostriatal pathways gate processing of behaviorally relevant reward dimensions
Source: PLoS Biol. 2018 Oct 19;16(10):e2005722. doi: 10.1371/journal.pbio.2005722 (PMC6209378; doi:10.1371/journal.pbio.2005722)
Supplement: S1 Table — (DOC) [file pbio.2005722.s001.doc]

**Table S1.** List of items used in the study.

| AA Batteries | Lip Balm |
| --- | --- |
| Alarm Clock | Massage Ball |
| Arts & Crafts Tool | Measuring Stick |
| Bike Carrier Rope | Napkins |
| Bike Repair Kit | Padlock |
| Candle | Pen Holder |
| Cheese Slicer | Pick Up Sticks Game |
| Clothes Hangers | Picnic Cups |
| Colored Pencils | Picture Frame |
| Cooking Utensils | Pizza Wheel |
| Flashlight | Playing Cards |
| Folding Travel Brush | Pocket Knife |
| Four Color Pen | Post-It Flag Highlighter |
| Gift Wrapping Paper | Shower Gel Duck |
| Hand Cream | Stamp Set |
| Hand Towel | String Lights |
| Herb Set | Sunscreen |
| Key Caps | Wooden Interlocking Puzzle |
| Kitchen Knife Set | Tea Strainer |
| Knife Sharpener | Tea Tin |
